# From symptom onset to care-seeking: a process-oriented mixed-methods study of prehospital delay in cerebral infarction

**DOI:** 10.3389/fpubh.2026.1823472

**Published:** 2026-07-03

**Authors:** Biao Xu, Zhi-Qiang Ge, Yi-Fan Shao, Cheng-Xiao Ma, Li Li

**Affiliations:** 1 School of Government, Nanjing University, Nanjing, China; 2 Department of Medical Office, Nanjing Drum Tower Hospital, Nanjing, China; 3 Department of Neurology, Nanjing Drum Tower Hospital, Nanjing, China

**Keywords:** health promotion, illness perception, mixed methods, patient delay, stroke

## Abstract

**Background:**

Cerebral infarction (ischemic stroke) is a highly time-sensitive acute cerebrovascular event, yet prehospital delays in patients’ accessing medical care remain prevalent across various countries and healthcare systems. Previous studies have primarily attributed delays to inadequate symptom recognition or barriers in healthcare accessibility, with limited systematic exploration of their formation mechanisms from a decision-making process perspective.

**Methods:**

This study integrated the Self-Regulation Model and the three-stage delay model, employing an explanatory sequential mixed-methods approach to examine the cognitive and decision-making processes among cerebral infarction patients who delayed seeking care. A total of 68 patients completed the Brief Illness Perception Questionnaire, and semi-structured interviews were conducted with 16 of these patients.

**Results:**

Findings indicate that delays in seeking medical care result from distinct mechanisms operating at different stages. During the appraisal delay stage, patients tended to normalize early or atypical symptoms. In the illness delay stage, optimism bias, self-regulatory overestimation, and doubts about medical efficacy reinforced a “wait-and-see” strategy. At the utilization delay stage, considerations of financial burden, family responsibilities, and societal role consequences significantly inhibited immediate healthcare-seeking behavior. Both quantitative and qualitative analyses revealed a cognitive-behavioral disconnect between illness perceptions and actual healthcare decisions.

**Conclusion:**

This study extends the explanatory power of Self-Regulation Model in healthcare decision-making contexts from a process-oriented perspective and offers a theoretical basis for designing staged, cognition-focused interventions aimed at reducing treatment delays.

## 1 Introduction

Cerebral infarction (ischemic stroke) is one of the leading causes of death and long-term adult disability worldwide, and its high morbidity, disability, and mortality rates make it a continuously increasing public health challenge (1, 2). Even if the patients survive, they are often left with varying degrees of neurological deficits, such as hemiparesis, aphasia, and cognitive deficits, which not only seriously affect the quality of life of the patients, but also impose a heavy burden of care and economic pressure on the family and society (3). Although recanalization therapies, such as intravenous thrombolysis and endovascular thrombolysis, have made significant progress in improving the prognosis of patients, these therapies are highly time-sensitive, and “time is of the essence” has become the core consensus of stroke care (4, 5). However, a persistent and unexplained dilemma is that even though cerebral infarction is widely recognized as a highly urgent, disabling, and even fatal condition, patient response to medical care after the onset of symptoms remains highly heterogeneous, and prehospital delays are still common (6).

Epidemiologic studies have shown that delays in access to care for patients with cerebral infarction are not confined to a specific country or healthcare system, but are a common phenomenon across regions and systems. In multi-country studies, including China, India, Thailand, and Morocco, the proportion of people arriving at the hospital more than 4.5 h after stroke onset generally ranged from 40 to 85% (7–10). Even in Switzerland, where the healthcare system is highly developed, significant prehospital delays are still found in about 42% of patients (6). A large body of evidence further confirms that delayed access to healthcare significantly increases the risk of death, readmission, and stroke recurrence in patients with cerebral infarction, with far-reaching detrimental effects on both acute-phase outcomes and long-term prognosis (1). These findings collectively indicate that delayed access to medical care is a persistent and high-risk public health issue, and its causes cannot be fully explained solely from the perspective of medical resources or technology.

Established research has explored the causes of delays in seeking medical care for patients with cerebral infarction on multiple levels. Sociodemographic and situational factors, such as rural residence, lack of witnesses at onset, and nocturnal or sleep onset, have been repeatedly shown to be significantly associated with longer delays (11, 12). In recent years, research perspectives have gradually extended to psychological and behavioral dimensions, and have begun to focus on the role of factors such as patients’ coping styles (e.g., avoidance, submission), personality traits, social support, and disease stigma in the decision to seek medical care (13, 14). Among these, “inadequate symptom recognition” is widely recognized as one of the central and most intervening factors contributing to prehospital delay (7, 15). However, a growing body of research evidence suggests that a simple emphasis on symptom recognition is difficult to adequately explain the complex decision-making process of delaying medical care, which involves multiple cognitive assessments and behavioral modifications. In real-world situations, even when patients are able to recognize physical abnormalities, especially when symptoms are atypical or initially mild, their decision to seek medical care is often accompanied by hesitation, waiting, and self-judgment (12, 16). Further studies have found that prehospital delays are not primarily comprised of transportation or healthcare system processes, but rather “decision-making delays” that occur after symptom onset and before the decision to seek care, accounting for nearly half of these delays in some studies (17). These findings suggest that delays in seeking care are not triggered by a single cognition, but are more likely to result from patients’ subjective understanding of the overall meaning of the illness and their decision-making process over time, and may lead to a disconnection between illness perceptions and actual care-seeking behavior.

From the perspective of the decision-making process, the three-stage delay model proposed by Safer et al. (18) suggests that the decision from symptom onset to treatment is not a one-time decision, but rather goes through three relatively independent but interrelated stages, namely, “appraisal delay,” “illness delay,” and “utilization delay,” which correspond to “am I sick,” “do I need professional medical help,” and “Is that care worth the costs”. The model provides an important structural framework for identifying the specific points at which delays occur. However, existing studies have mostly analyzed the overall delay time or focused on a single decision point, and less systematically examined the variability of patients’ cognitive mechanisms in different delay phases, thus limiting the in-depth understanding of the intrinsic logic of delayed behaviors. In this context, relying solely on stage delineation by itself still makes it difficult to answer a key question: why do patients make very different choices to wait or act at different stages when faced with similar symptoms? The Self-Regulation Model (SRM) provides an important theoretical perspective for understanding this issue. The model suggests that individuals form coping strategies and continuously regulate their behavior in the face of a health threat based on multidimensional cognitive representations of the disease (e.g., disease identity, severity, controllability, and disease duration expectations) and their affective responses (19). Therefore, introducing a SRM into the three-stage delay framework can help to reveal how disease perceptions in different decision-making stages shape patients’ subjective assessments of risks, benefits, and costs of action, thus explaining the possible disconnect between perceptions and healthcare-seeking behavior.

Based on this, this study integrated the SRM and the three-stage delay model, adopted an explanatory sequential mixed method design, used the Brief Illness Perception Questionnaire (BIPQ) (20) to quantitatively evaluate the multi-dimensional disease cognition of patients with cerebral infarction, and conducted an in-depth analysis of the cognitive logic and decision-making process in different delay stages through qualitative interviews. The theoretical contribution of this study is to go beyond the research paradigm that regards delay in medical treatment as a single behavioral outcome, and reveal the differential mechanism of disease cognition in different delay stages from the perspective of staged decision-making, thereby expanding the explanatory boundaries of the SRM in the context of acute disease medical treatment decision-making, and providing a theoretical basis for accurately positioning cognitively oriented early intervention.

## 2 Method

### 2.1 Study design

This study adopted an explanatory sequential mixed-methods design to systematically explore the role of disease cognition in the decision-making process of delayed medical treatment in patients with cerebral infarction. Delaying medical treatment is not a one-time behavioral choice, but a staged decision-making process that is constantly re-evaluated and adjusted as symptoms evolve, involving the dynamic interaction of cognitive judgment, emotional response and situational constraints. In this context, a single research method is insufficient to simultaneously reveal the overall characteristics and inherent decision-making logic of delayed medical care. Therefore, it is necessary to integrate quantitative and qualitative methods in an orderly manner to obtain a more comprehensive and in-depth understanding of this phenomenon.

A central feature of the explanatory sequential mixed-methods is the clear logical sequence and division of interpretive functions between research phases. In this design, quantitative research is conducted first to describe the overall characteristics of the research object and identify key patterns; qualitative research is subsequently carried out to contextualize and mechanistically explain findings that are difficult to fully explain or appear to be inconsistent in the quantitative results. Two stages form a continuous explanatory path in the research design: quantitative results are used to locate cognitive features that require further explanation, while qualitative analyzes are used to deepen the understanding of the mechanisms of action of these features in different delay stages. Through this orderly and connected mixed methods design, this study can not only describe the overall picture of disease cognition in patients with cerebral infarction, but also explain from a mechanistic level how disease cognition shapes delayed medical treatment behavior at different decision-making stages.

In this study, the purpose of the quantitative phase was not to conduct hypothesis testing, but to identify the distribution characteristics of disease cognition and its potential tensions, so as to provide explanation clues and focus for subsequent qualitative research. At this stage, the BIPQ is used to measure the multi-dimensional disease cognition of patients with cerebral infarction, answering questions such as “how patients generally view their disease”, “whether they think the disease is serious, controllable and whether the treatment is effective”, focusing on describing the overall cognitive characteristics of patients in terms of disease severity, controllability, disease course expectations and emotional reactions. Based on the quantitative survey, the research entered the qualitative stage to further answer questions such as “Why did these cognitions fail to be converted into medical treatment behavior when they entered the real situation?” “What changes have occurred in disease cognition during different decision-making stages such as assessment delay, disease delay, and use delay?” To ensure that the qualitative phase was systematically informed by the quantitative findings, the research team first conducted a preliminary analysis of the BIPQ data to identify key patterns requiring further explanation. The qualitative stage focused on patients’ subjective experience and decision-making narratives, and explored in depth through semi-structured interviews how patients understand and explain their symptoms in real medical situations, how they weigh disease risks and medical costs, and how these cognitive judgments are activated, modified or weakened at different stages from the onset of symptoms to the formation of medical decisions, thus affecting the formation and continuation of delayed medical treatment behavior.

### 2.2 Setting and procedure

This study was conducted from June to September 2025 in the neurology inpatient ward of a tertiary-level hospital in Nanjing, with study participants recruited during hospitalization by the research team. The research site is a regional general hospital, which is responsible for the acute diagnosis, treatment and hospitalization management of stroke patients. The patients received cover different age groups and clinical characteristics, and have certain population heterogeneity. Therefore, this hospital receives transferred patients from the three provinces of East China (Jiangsu Province, Anhui Province, and Zhejiang Province). The characteristics of the population are highly heterogeneous, and some patients are accompanied by more special or serious clinical conditions. As mentioned earlier, data collection was conducted first with a quantitatively oriented phase, followed by qualitative data collection through semi-structured interviews. In this study, “prehospital delay” was operationally defined as the time interval from the patient’s self-reported onset of first cerebral infarction-related symptoms to their arrival at a hospital for medical care. The study inclusion criteria include: ① age ≥18 years; ② clinically diagnosed with cerebral infarction and not seeking medical treatment within 24 h from the onset of symptoms; ③ having sufficient language understanding and expression skills to complete written questionnaires and participate in interviews; ④ voluntarily participate in the study and sign written informed consent. The quantitative sample was determined pragmatically based on the study period and patient availability, enrolling all eligible patients consecutively. The qualitative sample was guided by data saturation. This pragmatic approach to quantitative sampling and saturation-guided qualitative sampling is appropriate for exploratory mixed-methods research aimed at pattern identification and mechanism exploration. Among the participants who met the above criteria, the study used convenience sampling and invited some patients to participate in one-to-one in-depth interviews. The interviews focused on the patients’ overall perception of the disease, their subjective feelings after the onset of symptoms, as well as their coping styles after the onset of symptoms, and how they made judgments and decisions related to seeking medical care during the evolution of symptoms. To ensure the reliability of the interview data, the researchers took a detailed past medical history of the participants during the recruitment process, assessed whether they met the inclusion criteria, and excluded patients from the interview phase of the analysis if they had a clearly documented diagnosis of dementia or other cognitive impairment. For eligible patients who were not accompanied by a visitor or a healthcare team present at the time, researchers were invited in a face-to-face manner. As mentioned earlier, data collection began with a quantitative-oriented phase, followed by qualitative data collection through semi-structured interviews.

### 2.3 Quantitative data collection and analysis

In this study, the demographic and clinical characteristics of the participants were collected through a standardized case report form, including age, gender, literacy, income level, and major symptom manifestations. Based on the SRM, the BIPQ was used in this study to assess patients’ perceptions of illness. This scale has been widely used globally across different disease types and age groups with good concurrent validity (21, 22). The BIPQ contains 8 quantitative items covering multiple dimensions of cognitive and affective responses to illness. Among them, five items were used to assess the cognitive representation of the disease, including the perception of the consequences of the disease (item 1), the expectation of the duration of the disease (item 2), the perceived personal control (item 3), the awareness of the control effect of treatment (item 4), and the degree of identification with the disease (item 5). Another two items were used to assess the emotional representation, namely the degree of concern about the disease (item 6) and the intensity of emotional response (item 8). In addition, one item was used to measure the patient’s understanding of the disease (item 7). All quantitative items are scored numerically from 0 to 10, with higher scores indicating higher levels of the corresponding cognitive or affective dimension. For example, a higher score on the course of illness item indicates that the patient perceives the illness to have long-term or chronic characteristics, whereas a lower score on the personal control item reflects a weaker perception of his or her ability to control the illness. In addition to the quantitative items described above, the BIPQ contains an open-ended question to assess patients’ disease-causal perceptions, asking participants to list the three most important factors they believe contributed to the development of their illness. The research team classified the open-ended responses based on the nature of the perceived causes, such as demographic factors, genetic factors, lifestyle factors, and previous underlying diseases. Previous studies have shown that multiple dimensions of the BIPQ are closely related to important health outcomes. For example, high perceptions of disease consequences, strong disease identity, and significant levels of emotional reactions and concerns are often associated with higher levels of depression, anxiety, and poorer quality of life; while a higher sense of personal control is associated with lower psychological burden and better quality of life (21).

Quantitative data were statistically analyzed using SPSS 20.0 software. Demographic characteristics, clinical variables and questionnaire measurements were analyzed and presented by descriptive statistics.

### 2.4 Qualitative data collection and analysis

After completing the quantitative data collection, this study further conducted one-on-one semi-structured in-depth interviews to obtain richer information related to delayed medical treatment in patients’ medical experience. The purpose of the qualitative interviews was to further explain the responses of the research subjects in the BIPQ, and to gain a deeper understanding of how and why individuals respond to symptoms during the onset and exacerbation of symptoms, and why they delay seeking medical treatment.

The interview adopted a semi-structured interview outline, consisting of open-ended questions, focusing on the patient’s understanding of his or her disease, subjective feelings about symptoms, and the specific decision-making process from symptom onset to final admission. The core interview questions mainly include: ① Why did you feel uncomfortable and were admitted to the hospital this time? ② Do you know about this disease? ③ What was your specific experience from the onset of symptoms to admission to hospital? These questions relate to how individuals understand their illness, how they feel about the illness, and how they make medical-seeking-related judgments about symptom exacerbation based on a SRM, revealing the role of illness cognition in decision-making to delay medical treatment. The interviews were conducted by two team members, both of whom had previous experience in qualitative research and had no prior relationship with the interviewees. The interviews were mainly conducted face-to-face in a private confined space in the hospital to protect the privacy and freedom of expression of the interviewees. Each interview lasted an average of 20 min, and was audio-recorded, transcribed verbatim, and text-analyzed and coded using NVIVO 12.0.

Qualitative data collection and analysis occurred simultaneously. The determination of data saturation is based on a full understanding of the connotation of coding, rather than simply relying on the number of codes. It is marked by the fact that new dimensions or new meanings of existing themes are no longer identified in the ongoing analysis (23–25). To operationalize this process, two members of the research team independently coded the transcript using NVivo 12.0 following each interview. The team convened weekly to compare codes, resolve discrepancies through discussion, and review the codebook for emerging themes. Saturation was systematically assessed after every three interviews and was deemed to be approached when no new codes emerged across three consecutive interviews. When the research team encounters the aforementioned situation during the analysis process, further recruitment of interviewees is stopped. Saturation occurred after 14 interviews. Two additional interviews were subsequently conducted to confirm that no new information had emerged. This number of interviews is consistent with general recommendations in the field of health research that data saturation is typically achieved after approximately 9–17 in-depth interviews in contexts that focus on the research topic (23).

### 2.5 Data integration

This study achieved systematic integration of quantitative and qualitative data in a multi-level approach to fully leverage the advantages of an explanatory sequential mixed-methods design. First of all, the integration strategy of “following a thread” was adopted during the research process, which used the key findings identified in the BIPQ quantitative questionnaire as clues to guide the in-depth development of subsequent interviews. During the qualitative interviews, research subjects were encouraged to further explain and reflect on their responses to the BIPQ, so that the quantitative results could be interpreted in a specific context. This process helps orientate the research phenomenon prior to qualitative analysis and establish progressive relationships between different data sources. Secondly, in the results presentation stage, the study constructed a joint display table (joint display) to classify the relevant factors identified in the interviews and affecting medical decision-making according to the semi-independent decision-making stages such as appraisal delay, illness delay and utilization delay, and compared them with the corresponding cognitive dimensions in the concise disease perception questionnaire. Through this juxtaposed presentation, results from different research methods can be compared under the same analytical framework, thereby enhancing the overall understanding of the decision-making mechanism of delaying medical treatment. Finally, in the discussion stage, quantitative and qualitative research findings were comprehensively integrated, and from the perspective of staged decision-making, the role of disease cognition in different delay stages was systematically sorted out. Through explanatory integration across stages and methods, this study formed a comprehensive understanding of the medical help-seeking behavior of patients with cerebral infarction.

## 3 Results

### 3.1 Participant characteristics

During the BIPQ questionnaire survey stage, 68 participants were recruited (Table 1). Their mean age was 68 ± 12 years (range: 43–91 years), and the majority were male (75%). Participants had an average of 5 ± 2 comorbidities, primarily including hypertension, diabetes, and heart disease. The median delay time was 10 days.

**Table 1:** Participant sociodemographic and clinical characteristics variables (*N* = 68).

Participant characteristics	Mean ± SD or *n* (%)
Demographics	
Age (years)	68 ± 12
Male	51 (75%)
Admission method	
Voluntary admission	29 (42.6%)
Family recommendations	33 (48.5%)
Medical recommendations	6 (8.8%)
Main symptoms	
Weakness/numbness of limbs	42 (61.8%)
Dizziness/headache	16 (23.5%)
Unclear speech	10 (14.7%)
Orofacial tilt/facial paralysis	4 (5.9%)
Total number of co-morbidities	5 ± 2
Annual income	
No income	30 (44.1%)
<10,000 RMB	20 (29.4%)
10,000–50,000 RMB	12 (17.6%)
60,000–100,000 RMB	4 (5.9%)
>100,000 RMB	2 (2.9%)
Education level	
None	22 (32.4%)
Primary school	20 (29.4%)
Junior high school	12 (17.6%)
High school	10 (14.7%)
Bachelor degree and above	4 (5.9%)

RMB, Renminbi.

### 3.2 Illness perceptions

In the survey results of the BIPQ, the median score of participants’ perception of the helpfulness of current treatment (item 4) was 8 (see Table 2), which shows that most patients believe that the existing treatment options are highly effective in disease management, which reflects the timeliness and pertinence of medical interventions after actual admission, and patients have a high degree of trust in the treatment options. The median score for disease concern (item 6) was also 8, indicating that participants generally maintained a high level of concern about their disease status, and some patients even showed extremely high levels of worry. This may be related to the severity of symptoms of the disease (such as limb weakness, language impairment, etc.) or uncertainty about long-term prognosis, which is consistent with the findings on disease duration (item 2). Regarding the perception of disease duration (item 2), participants overall believed that the disease had a long duration. The median score for the perceived impact of the disease on daily life (item 1) was 7, indicating that the disease had a moderate to high impact on daily life. Regarding sense of personal control (item 3), the median score was 6, indicating a moderate level of participants’ perception of their ability to control their disease. In terms of emotional representation, the median score of the disease’s impact on emotions (item 8) is 6, but individual differences are obvious. About half of the patients suffer from moderate emotional distress, and some patients (Q1 = 3) have less emotional impact. The median score for understanding of disease (item 7) is 5, indicating that there is still much room for improvement in health education, and some patients may lack a clear understanding of disease mechanisms, prognosis and management strategies.

**Table 2:** Results of the BIPQ (*N* = 68).

BIPQ item	Median score (IQR)
Consequences	
Item 1. How much does your illness affect your life?	7 (6, 8)
Timeline	
Item 2. How long do you think your illness will continue?	5 (3, 7)
Item 3. How much control do you feel you have over your illness?	6 (5, 7)
Treatment control	
Item 4. How much do you think your treatment can help your illness	8 (7, 9)
Identity	
Item 5. How much do you experience symptoms from your illness?	7 (6, 8)
Concern	
Item 6. How concerned are you about your illness?	8 (6, 9)
Comprehensibility	
Item 7. How well do you feel you understand your illness?	5 (3, 7)
Emotional response	
Item 8. How much does your illness affect you emotionally?	6 (3, 7)

In terms of cognition of disease causation, participants listed a total of 21 factors that they believed were related to the occurrence of the disease (see Table 3), reflecting a certain heterogeneity in cognition of the causes of cerebral infarction. The most commonly mentioned factors include: (1) underlying diseases such as high blood pressure or hyperglycemia; (2) lifestyle factors such as smoking and drinking; and (3) age or genetic factors. To present an overall trend, the study classified the above responses into five categories. The results show that most patients (75%) believe that underlying diseases (such as hypertension, diabetes) are the most important causative factors, which is consistent with medical research; followed by (56%) are unhealthy lifestyles (such as smoking, drinking), indicating that some people are aware of modifiable behavioral risks. Older patients pay more attention to age factors, while younger patients place more emphasis on genetics or lifestyle.

**Table 3:** Classification and frequency of disease attribution (*N* = 68).

Category	Contributing factor	Frequency
Underlying disease comorbidities	Hypertension, diabetes mellitus (hyperglycemia), hyperlipidemia, heart disease (including coronary heart disease, atrial fibrillation)	54
Unhealthy habits (adjustable)	Smoking, drinking, late nights, poor diet, lack of exercise, poor weight management, poor sleep	35
Unadjustable factors	Age (old age/aging), heredity (family history), genes, immunity	24
Psychological and emotional factors	Emotions, stress, mentality, mood	16
Occupational and environmental factors	Work (exertion/lifting iron), climate	3

This study supplemented correlation analysis, subgroup comparisons, and exploratory regression analysis based on descriptive statistics. Spearman correlation analysis revealed no significant correlations between delay time and any dimension of the BIPQ (all *p* > 0.05); however, “Disease understanding” (Item 7) exhibited a marginally significant positive correlation trend (r = 0.228, *p* = 0.061) (Appendix Table 2A). Patients were categorized into a short delay group (≤7 days, *n* = 37) and a long delay group (>7 days, *n* = 31), using a median of 7 days as the cutoff. The Mann–Whitney *U* test indicated no statistically significant differences between the two groups across all eight dimensions of the BIPQ (all *p* > 0.05) (Appendix Table 2B), and the results of the sensitivity analysis, using 15 days as the boundary, were consistent. A multiple linear stepwise regression analysis, with Log delay time as the dependent variable and eight dimensions of the BIPQ as independent variables, revealed that only Item 7 (disease understanding) was included in the final regression model (*F* = 4.199, *p* = 0.044, adjusted *R*^2^ = 0.046) (Appendix Table 2C). The standardized regression coefficient *β* = 0.245 (*p* = 0.044) suggests that greater patient understanding of the disease is associated with longer delay times. This analysis indicates that among the various dimensions of the BIPQ, only “disease understanding” demonstrates a weak positive predictive relationship with delay time, and the model’s explanatory power is relatively low. These findings confirm the existence of a “cognitive-behavioral disconnection” phenomenon in patients with cerebral infarction who postpone seeking medical treatment, implying that disease perception at a single time point inadequately captures the complexities of the phased medical decision-making process. Further investigation into its underlying mechanisms through qualitative interviews is warranted.

### 3.3 Interview findings

One-on-one semi-structured interviews with 16 of the participants who completed the questionnaire systematically revealed how patients with cerebral infarction understand their disease, what kind of emotional reactions they have, and how these disease perceptions gradually shape their coping styles and contribute to delayed medical treatment after the onset of symptoms. The interview results focused on the following aspects: patients’ diverse explanations for the cause of the disease; the logic behind the generally low sense of personal control and its impact on symptom response; the cognitive and emotional basis behind the high level of disease concern; the perception of the consequences of cerebral infarction (generally considered serious and with long-term effects); and the patient’s relatively weakened or delayed perception of emotional impact.

The interviewees were all patients who were hospitalized for the first time or again due to cerebral infarction. Most of them had comorbidities such as hypertension and diabetes, and there were significant differences in their past health experiences and symptom experiences, providing an important background for the heterogeneity of disease cognition. In the interview sample, 68.75% were male, the average age was 67 years old, 37.5% had no annual income, 31.25% had an annual income of less than 10,000 yuan, and 62.5% had an education of elementary school or less, as shown in Table 4. The symptoms of hospitalization were diverse, with common manifestations such as limb weakness, numbness, dizziness, crooked mouth, and slurred speech. The delay time varied widely, with the shortest being 6 h (P07) and the longest being up to 1 month (e.g., P03, P10, P14, P16), and the majority of patients were delayed for a few days to a few weeks. The length of the interview ranged from 16 min 57 s to 25 min 45 s, with an average of about 20 min. Regarding the mode of admission, seven patients were admitted “on their own initiative” and nine patients were admitted “on the advice of family members” or “on the advice of a doctor”, indicating that family and social support play an important role in decision-making about medical care.

**Table 4:** Basic information about the interviewees (*N* = 16).

Number	Gender	Main symptoms upon admission	Delay time	Interview duration	Admission method
P01	Male	Ptosis of the left eyelid with tilting of the corners of the mouth	3 days	21 m 45 s	V
P02	Female	Dizziness with headache	About 10 days	25 m 45 s	V
P03	Female	Weakness in the left limb	1 month	20 m 52 s	V
P04	Male	Weakness of the right side of the limb with slurred speech	3 days	29 m 25 s	F
P05	Male	Numbness of the right side of the mouth, lips and tongue	10 days	16 m 57 s	V
P06	Male	Numbness of the left limb	20 days	19 m 45 s	F
P07	Male	Weakness of the right side of the limb with slurred speech	6 h	21 m 14 s	F
P08	Male	Weakness in the left limb	13 h	19 m 6 s	V
P09	Male	Dizziness	2 days	20 m 7 s	V
P10	Male	Head and neck discomfort, left upper limb weakness	1 month	19 m 26 s	F
P11	Female	Abnormal movement, weakness of left limb	1 week	18 m 46 s	F
P12	Female	Weakness in the right lower limb and inability to walk	5 days	20 m 4 s	M
P13	Male	The right upper limb is numb and weak, unable to lift objects	1 week	20 m 28 s	F
P14	Male	Sluggish reactions and memory loss	1 month	18 m 2 s	F
P15	Male	Facial paralysis, left limb weakness	Half a month	19 m 26 s	F
P16	Female	Numbness and weakness of left limb	1 month	20 m 15 s	V

Admission method: V refers to “Voluntary admission”, F refers to “Family recommendations”, M refers to “Medical recommendations”.

The interviewees were from three provinces in China’s Yangtze River Delta region, which is a developed economic region in China with high accessibility to healthcare resources, and their delayed healthcare behaviors were generally attributed in the interviews to subjective judgments at the symptom assessment and decision-making stages, rather than to simple transportation or healthcare accessibility constraints.

Overall, the interview results are highly consistent with the SRM at the level of core theoretical assumptions. Therefore, the results are organized and presented around the different dimensions in the SRM of illness behavior and are further combined with the three-stage delay model to systematically present how illness perceptions play a differential role in the different decision-making phases of assessment delay, illness delay, and use delay.

#### 3.3.1 Insufficient awareness of disease

##### 3.3.1.1 Misidentification of symptoms of cerebral infarction: “normalizing” abnormal experiences

Interviews revealed that many patients failed to recognize the typical manifestations of cerebral infarction as abnormal signals requiring urgent treatment in the early stages of symptoms. Numbness or weakness in the limbs is often attributed to ordinary causes such as exertion and cold; slurred speech is interpreted as heatines or oral problems; unsteady walking is simply regarded as “bad legs and feet due to old age.” This misjudgment stems from the lack of knowledge of the disease and the progressive nature of the symptoms.

“I thought it was numbness in my hands and feet. If anyone mentioned the word cerebral infarction to me, I would never move. Later I found out that half of my body could not move, so I panicked.” [Participant 8, male].

“I feel like I’m tired from the work. Raising crabs... I just need to rest for a few days and it’ll be fine.” [Participant 15, male].

In the process, symptoms are progressively incorporated into a continuous spectrum of prior bodily experience, and their anomalousness is diminished, thus interrupting further risk assessment and decision-making about medical care.

##### 3.3.1.2 Underestimation of the harmfulness of the disease: treating cerebral infarction as an “affordable problem”

In addition to the lack of symptom recognition, patients generally lack adequate knowledge of the potential serious consequences of cerebral infarction, especially in rural areas and in populations with a low level of education. Many patients regard cerebral infarction as a “common disease of the older adult(s)” or a “minor problem that will get better slowly”, failing to realize that it may lead to irreversible consequences such as paralysis, aphasia and even death. And there are some older adult(s) patients, influenced by traditional cultural beliefs, believe that life will inevitably end and adopt a nonchalant attitude.

“Cerebral infarction may lead to paralysis? Oh...at first I thought it would get better slowly” [Participant 3, female].

“I’m 80 years old, people will eventually die when they’re old. This disease is not a big deal.” [Participant 1, male].

This systematic underestimation of the dangers makes patients tend to delay action even if they are aware of physical abnormalities. The delay is already formed during the assessment stage.

#### 3.3.2 Cognitive bias

##### 3.3.2.1 Expectation that symptoms will resolve spontaneously: optimism bias

Patients with cerebral infarction generally have a strong expectation that symptoms will resolve on their own. This cognitive bias is mainly manifested in an overestimation of self-regulation ability and is an important factor leading to delays in seeking medical treatment. Interview results showed that 68.7% of patients had a “wait and see” mentality in the early stages of symptoms, which mainly manifested into three types: the transient symptom experience misleading type, that is, some patients believed that the symptoms would naturally improve because they had experienced similar transient symptoms (such as transient ischemic attack) in the past; the self-regulation attempt type, patients tended to observe the effect through self-intervention measures such as rest and medication; the symptom fluctuation misjudgment type, because the symptoms of cerebral infarction often progress in a fluctuating manner, patients strengthen their expectations of self-healing when symptoms are temporarily relieved.

“I initially thought that taking a rest would make me feel better, but I delayed it for a month.” [Participant 11, female].

“Intravenous fluids would probably be fine. only to find out later that it was getting worse.” [Participant 2, female].

In this process, brief relief of symptoms is misinterpreted as a signal of recovery, which instead reinforces delayed behavior.

##### 3.3.2.2 Doubt of medical efficacy: confirmation bias

The interviews also revealed that some patients have systematic doubts about the value of formal medical care. Once patients form a preliminary opinion that “the role of medical treatment is limited” or “there is over-treatment in hospitals,” they are more likely to systematically question professional medical advice based on previous scattered medical experiences or negative information spread in society. Specifically, patients often interpret doctors’ recommendations for further examination or referral as “making a fuss out of a molehill” or profit-seeking behavior rather than professional judgment based on disease risks, thereby reducing their trust in standardized medical care and thus not going to regular hospitals for treatment, and instead rely on self-regulation or informal treatment methods, such as diet therapy or infusions that completely replace drugs and further diagnosis and treatment.

“The county hospital said I needed to be transferred to a higher-level hospital for treatment immediately. I thought they were making a fuss out of a molehill, so I went to a small clinic for intravenous infusion for three days.” [Participant 4, male].

“After the MRI was done, I was asked to do a CT angiography. I think the hospital just wants to make more money” [Participant 7, male].

As a result of this confirmation bias, when symptoms are not relieved as expected, skepticism about the efficacy of medical treatment is further reinforced, and self-regulation or informal pathways continue to be followed, ultimately leading to a cyclical decision-making pattern of “worsening symptoms - deepening skepticism - increasing delays”.

#### 3.3.3 Pressure of economic and family responsibilities

##### 3.3.3.1 Medical expense concerns

The delay in seeking medical treatment due to financial pressure in patients with cerebral infarction is a multi-dimensional problem. People without medical insurance or with unstable incomes often delay seeking medical treatment due to financial pressure. First, groups that lack stable income and social security (such as farmers and the older adult(s) without pensions) face difficulties in paying direct medical expenses, including direct costs such as examination fees, medication fees, and hospitalization deposits. Secondly, patients also need to consider indirect economic losses, such as income interruption during hospitalization and missed work expenses for accompanying family members. What is even more complicated is that the long-term rehabilitation treatment and repeated reexamination required for cerebral infarction bring about continuous financial burdens, causing many patients to choose the coping strategy of “Delay minor illnesses and endure major ones”. This kind of economic pressure creates a vicious cycle of “illness-poverty” among low-income families. Patients are often forced to seek medical treatment only after their symptoms seriously affect their ability to work. At this time, the best opportunity for treatment has been missed, and instead they have to bear higher medical expenses.

“I am a crab farmer. If I am hospitalized, who will do the work? What about the medical expenses?” [Participant 15, male].

“Rural people don’t have pensions, so they can carry it if they can.” [Participant 9, male].

##### 3.3.3.2 Family role conflict

In terms of family responsibilities, patients often need to balance their own health and family roles. During interviews, it was found that a higher proportion of patients postponed treatment due to taking care of family members or not wanting to trouble their children. Patients who are the main caregivers of their families (such as supporting the older adult(s) and raising grandchildren) often put the needs of their families before their own health, and prioritize ensuring the functioning of the family even if they have obvious symptoms. At the same time, older adult(s) patients who rely on their children for care have a strong “not causing trouble” mentality and conceal their illness to avoid affecting their children’s work or life arrangements. This role conflict is particularly prominent in multi-generational families with close intergenerational relationships. Patients often need to balance the opinions and interests of different family members, which prolongs the decision-making process and leads to delayed medical treatment.

“Mom was so sick no one was there to take care of her, I dragged myself dizzy for 20 days before I came.” [Participant 16, female].

“My son has a busy driving job and I don’t want to hold him back.” [Participant 12, female].

#### 3.3.4 Socio-cultural influences

##### 3.3.4.1 Non-professional misleading opinions

Non-professional advice is misleading and reinforces a wait-and-see attitude. Advice provided by some patients’ friends and relatives based on their limited medical knowledge often reinforces patients’ wait-and-see attitude. The influence of such non-professional advice stems from patients’ natural trust in their social circle and the possible lack of primary health education. When patients receive both “no need to go to the doctor” advice from friends and relatives and early warning signals from their bodies, social acceptance often overrides health rationality, leading to delayed diagnosis. This phenomenon is particularly prevalent in rural areas and among lower social class groups, reflecting structural differences in the dissemination and acquisition of medical knowledge.

“My neighbor advised me that ‘it’s natural to have more problems as you get older, so there’s no need to rush.” [Participant 14, male].

##### 3.3.4.2 Stigma and negative fatalism

Influenced by traditional cultural concepts, the common stigma and fatalistic outlook of patients with cerebral infarction constitute important obstacles to seeking medical treatment. On the one hand, some patients regard neurological dysfunction as a personal defect and form a hidden shame in their hearts, fearing the loss of social (family) value after diagnosis. On the other hand, some patients attribute the disease to natural aging or fateful destiny factors such as irresistible factors, such as traditional concepts such as “the end of life”, which is particularly prominent among older adult(s) patients. This mentality leads patients to deliberately avoid diagnosis and treatment to avoid being labeled as a “sick person” or a “family burden.” On a deeper level, it reflects traditional culture’s stigmatizing perception of disability and the lack of concepts of quality of life.

“People are always going to die one day sooner or later, this is no big deal......” [Participant 1, male].

### 3.4 Integration

The interview findings showed that patients had different forms of delayed behavior in each of the three decision-making stages: appraisal delay, illness delay and utilization delay, and that multiple cognitive and situational factors intersected at different stages. To systematically present this process, this study integrated quantitative findings with interview findings to construct a comprehensive outcome matrix (see Table 5), which mapped patients’ subjective experiences against the dimensions of the BIPQ questionnaire and summarized them by delay stage, thus revealing how illness perceptions shape delayed medical care behaviors at different decision-making nodes.

**Table 5:** Integrated results matrix.

Stage of delay	Corresponding decision	Characteristics of experience that influence decision making as revealed by interviews	Corresponding aspect of illness perception assessed by the BIPQ
Appraisal delay (Am I ill?)	Do symptoms require medical intervention?	*Misidentification of symptoms of cerebral infarction*: Mistaking specific symptoms (such as numbness in limbs, speech disorders) for common problems (for example, “thinking it’s a cold or a sprained muscle and bone”). *Lack of awareness of the dangers of the disease*: lack of understanding of the risk of disability/death from cerebral infarction (such as “I am 80 years old and will die sooner or later”).	*Identity*: How much do you experience symptoms from your illness? −7 (6,8) *Cause*: what do you think causes your symptoms? (top 3) –Underlying Disease Comorbidities –Unhealthy habits –Unadjustable factors
Illness delay (Do I need professional care?)	Is immediate medical attention required?	*Expectation that symptoms will resolve on their own (optimistic bias)*: Belief that rest can heal itself (e.g., “I initially felt that I would be fine just by resting, but I delayed it for a month”). *Medical efficacy doubt (confirmation bias)*: More trust in non-professional advice (such as “A friend said that intravenous drip is enough for high blood pressure”). *Family role conflict*: Due to delays in taking care of family members (such as “My mother was seriously ill and no one was looking after her, and I was dizzy myself and put it off for 20 days”).	*Cause*: what do you think causes your symptoms? (top 3 as above) *Consequences*: how much does your illness affect your life? − 7 (6, 8) *Personal control*: how much control do you feel like you have over your illness? −6 (5, 7) *Concern*: How concerned are you about your illness?-8 (6, 9)
Utilisation delay (Is this care worth it?)	Do the benefits of access to health care outweigh the costs?	*Medical expense concerns*: Delayed due to economic pressure (such as “Rural people have no pensions, so they should bear it if they can”). *Misleading advice from relatives and friends*: Strengthen the wait-and-see attitude (for example, “Neighbors advise that as one gets older, they have more problems and there’s no need to rush”). *Stigma of illness and negative fatalism*: Attributing illness to fate (such as “Everyone has to die, it makes no difference whether they are treated or not”).	*Consequences*: how much does your illness affect your life? −7 (6, 8) *Timeline*: how long do you think your illness will continue? −5 (3, 7) *Treatment control*: how much do you think treatment can help your illness? −8 (7, 9)

The values in each disease perception dimension of BIPQ are represented as: median score (IQR).

Further integration of the quantitative and qualitative results revealed both convergence and divergence across the three stages of delay. Regarding convergence, the quantitative finding that “illness comprehensibility” (Item 7) independently predicted longer delay corresponded with the qualitative theme of symptom normalization during the appraisal delay stage. During this stage, patients often interpreted early symptoms as normal aging or fatigue, as illustrated by the statement, “I thought resting would make it better.” In terms of divergence, the quantitative analysis indicated weak correlations between BIPQ dimensions and delay time. The qualitative interviews elucidated this divergence by revealing stage-specific mechanisms: optimism bias and self-regulation overestimation predominated during illness delay, while financial pressure, family role conflicts, and fatalism were more influential during utilization delay. This divergence implies that single-timepoint illness perceptions cannot accurately capture prehospital delay, as different cognitive and situational factors influence decision-making at various stages, thereby underscoring the necessity of a staged decision-making framework.

## 4 Discussion

This study offers several notable contributions. It tackles prehospital delays in ischemic stroke by innovatively integrating the Self-Regulation Model with the three-stage delay framework. The explanatory sequential mixed-methods design exhibits methodological rigor, as quantitative findings inform qualitative inquiry, with data saturation meticulously achieved. The qualitative data clarify stage-specific mechanisms—appraisal, illness, and utilization delays—that quantitative analysis alone cannot elucidate. These findings yield actionable, phase-targeted intervention strategies for clinicians and policymakers.

### 4.1 Delay in symptom perception and assessment

Consistent with existing research on delayed medical treatment in acute illnesses (7, 26, 27), this study found that patients with cerebral infarction generally have difficulty identifying the abnormality of physical changes in the early stages of symptom onset, especially when symptoms develop gradually or present in atypical forms. Interview results show that typical symptoms of cerebral infarction, such as numbness of limbs, weakness and slurred speech, are often interpreted by patients as fatigue, cold or part of natural aging. This cognitive pathway of “normalizing” abnormal symptoms prevents patients from promptly defining body signals as health threats requiring urgent medical intervention, thereby significantly extending the evaluation delay phase.

This finding echoes the low median BIPQ “disease understanding” score (5 points) in the quantitative results, suggesting an overall lack of awareness among patients of the symptom spectrum of cerebral infarction and its potentially serious consequences. This lack of cognition is not simply a lack of information, but more like an active evaluation process based on existing experience: patients tend to use common sense in life, previous health experiences, and cultural frameworks to reinterpret emerging body changes and bring them back into the “acceptable normal range”, thus suspending further action decisions.

In addition, repeated underestimation of the harmfulness of the disease in the interviews (such as considering cerebral infarction as a “common problem of the older adult(s)” or “a natural result of aging”) further reinforced the delay in assessment. Such explanations not only downplay the urgency of the symptoms but also reduce the patient’s perceived risk of potentially serious consequences, such as paralysis, aphasia, or death. This result suggests that in existing health education, simply emphasizing symptom recognition may not be enough. It is more necessary to systematically strengthen the public’s understanding of the seriousness of the consequences of cerebral infarction and its “interventionability” to break the cognitive cycle of rationalizing abnormal symptoms and delaying action.

### 4.2 Cognitive representation and emotional response

During the delayed stage of illness, patients’ cognitive representations and emotional responses jointly shape their coping styles. This study found that optimism bias and overestimation of self-regulation ability are prevalent in most patients with cerebral infarction, which are manifested in coping strategies such as “watch again” and “it may be relieved after resting.” This phenomenon is consistent with the moderate level of the “personal control” dimension score in the BIPQ (median 6 points), suggesting that patients are not completely passive or indifferent to the disease, but tend to incorporate symptoms into self-manageable categories. This finding is consistent with the basic assumption of the SRM, which is that when individuals face health threats, they often first restore the “normal state” through cognitive evaluation and self-regulation attempts, rather than immediately seeking external intervention.

However, existing studies have pointed out that self-regulation does not always have a protective effect in acute or rapidly progressing disease situations (26, 28). This study further shows that in the highly time-sensitive situation of cerebral infarction, this kind of cognitive representation centered on “self-controllability” may actually conceal the risk of disease progression. When symptoms do not relieve as expected, some patients do not re-evaluate the severity of the disease or turn to professional medical care, but continue to strengthen self-regulation or alternative coping methods. This process was often accompanied by systematic questioning of medical efficacy during interviews. Specifically, patients develop distrust in further examinations, referrals or standardized treatment recommendations based on past scattered medical experiences or negative information spread in society, interpreting them as “over-medical treatment” or profit-seeking behavior. This reaction can be understood as a typical confirmation bias: patients tend to choose information and action paths that are consistent with their existing beliefs (“symptoms are not serious” and “no need for major hospital intervention”), while avoiding or denying medical judgments that conflict with them. As a result, self-regulation failed to trigger a transition to professional care, and instead formed a cycle of “recurrence of symptoms—intensification of suspicion—deepening of delay”.

This finding is highly similar to the “cognitive dissonance-avoidance coping” mechanism proposed by Ivynian et al. (29) in patients with heart failure. The study points out that when patients are unable to match their status with the identity of a “severely ill patient,” they often relieve the resulting emotional stress by denying, downplaying, or avoiding it. This study extended this mechanism to the context of cerebral infarction and further found that this avoidance does not necessarily manifest as strong emotional distress. On the contrary, some patients did not report a high level of emotional response in the scale, but showed obvious procrastination and avoidance behaviors during the interview. This inconsistency of “low reported emotion - high avoidance behavior” suggests that relying solely on scales may underestimate the indirect role of emotion in delaying medical treatment, while emotion plays more of an impact by shaping cognitive defenses and coping strategies. This “cognitive-behavioral disconnect” is further corroborated by recent studies (30–32), which consistently report that symptom recognition alone does not translate into timely care-seeking and that “wait-and-see” behaviors are prevalent across different cultural contexts.

### 4.3 Social consequences and utilization delay

In the delayed stage of utilization, financial pressure, family responsibilities and social and cultural factors constitute important external constraints that affect medical treatment decisions. Although quantitative results showed that patients’ overall perceptions of treatment effectiveness were high (median BIPQ “Control of Treatment” dimension score was 8), interview results showed that this recognition of the value of care did not necessarily translate into actual care-seeking behavior. This “cognition-action disconnect” phenomenon is highly consistent with the inhibitory effect of structural constraints on healthy behaviors repeatedly pointed out in existing research, especially among groups with limited resources or greater social responsibility pressure. First, financial stress has been repeatedly shown to be an important predictor of delayed medical care, including both direct medical expenditures and the ongoing financial burden of income interruption during hospitalization and subsequent long-term recovery (33, 34). This study found that patients with low income or lack of social security, especially farmers and older adult(s) without retirement security, often choose the strategy of “putting it off as long as they can” before their symptoms seriously affect basic life functions. This behavioral logic does not stem from the recognition of the ineffectiveness of medical treatment, but is based on rational expectations of “long-term unbearable costs”, which is consistent with the decision-making model of “delay to avoid future uncertain losses” in health economics. Second, family role conflict plays a key role in delayed use. Interviews show that patients, who are the main caregivers of their families, tend to give up their own health needs to family responsibilities; while older adult(s) patients generally show a reluctance to “cause trouble for their female children.” This concern about “becoming a burden to others” is highly consistent with the social consequence orientation proposed in existing research, that is, individuals not only weigh health benefits when making medical decisions, but also systematically evaluate the impact of medical behaviors on family members, care relationships, and social roles (29). Under this framework, medical treatment is no longer a pure health behavior, but is perceived as an event that may destroy family balance and personal social value, and is therefore delayed or even avoided. In addition, the fatalistic tendencies and stigma displayed by some patients during interviews further exacerbated the delay in use. This cultural orientation is particularly evident among older adults, where disease is understood to be the result of natural aging or destiny rather than a health event that can significantly change the prognosis through timely medical intervention. Existing research has shown that fatalistic beliefs and stigma can weaken an individual’s perception of the controllability of medical intervention and reduce their willingness to actively seek professional help, especially in diseases with a high risk of neurological damage or potential disability (35, 36). The findings of this study further indicate that in the highly time-sensitive situation of cerebral infarction, this cultural sense of “irreversible cognition” may cause patients to choose to give up immediate action even if they recognize the effectiveness of treatment.

## 5 Theoretical contributions

This study systematically expands the decision-making mechanism of patients with cerebral infarction to delay medical treatment from a theoretical level, and responds to the deficiencies of existing research in process characterization, mechanism explanation, and theoretical extrapolation, which are mainly reflected in the following three aspects:

First, at the research paradigm level, this article reconstructs the analytical framework of delayed medical treatment in patients with cerebral infarction from a process perspective. In view of the fact that most existing studies simplify delayed medical treatment to a single time indicator or final behavioral result, thereby ignoring the limitations of its internal decision-making evolution process. This article redefines delayed medical treatment as a staged decision-making process consisting of symptom assessment, disease judgment, and medical use. By introducing and refining the three-stage delay model, this study systematically depicts the formation logic and inherent differences of delayed medical treatment at different stages. It provides a clear process-oriented analysis framework for understanding “how delay is generated and gradually solidified” in cerebral infarction, a highly time-sensitive disease, thus promoting the theoretical shift in delayed medical treatment research from outcome-oriented to process-oriented.

Second, at the mechanism level, this article reveals the generative mechanism of delaying medical treatment in different decision-making stages, which is jointly driven by cognitive evaluation, emotional regulation and consideration of social consequences. Different from existing studies that mainly explain the path of delayed medical treatment from insufficient symptom recognition or medical accessibility barriers, this article found that the delayed behavior of patients with cerebral infarction stems from the dynamic interaction of multiple psychological and social mechanisms. In the assessment and disease delay stages, cognitive mechanisms such as symptom normalization, optimism bias, and confirmation bias often reinforce each other with emotional regulation methods such as denial and avoidance; while in the use delay stage, systematic assessment of financial burdens, family responsibilities, and loss of social roles further reshapes patients’ subjective weighing of the costs and benefits of medical treatment. This finding shows that delayed medical treatment is not the direct result of a single psychological or structural factor, but the coupling product of multiple cognitive and emotional mechanisms in specific social situations, thus deepening the theoretical understanding of the logic of delayed medical treatment.

Third, at the level of theoretical expansion, this article makes a procedural revision to the disease cognition theory in explaining acute medical seeking behavior by revealing the phenomenon of “cognition-behavior disconnection.” Existing disease perception research generally assumes that higher disease concern and trust in treatment effectiveness directly promote timely medical treatment. However, this study found that even if patients show a high sense of treatment control and disease concern at the scale level, their actual medical treatment behavior may still be significantly delayed. This “cognition-behavior disconnect” phenomenon shows that disease cognition does not automatically translate into action, but requires situational assessment and social meaning construction. This article clarifies the specific links and conditions that block this transformation through staged analysis, thereby introducing a more explanatory process perspective and situational dimension to the disease cognitive theory, and expanding its explanatory boundaries for the complexity of acute health decision-making.

## 6 Realistic enlightenment

The findings of this study have significant implications for clinical practice, public health interventions, health communication, and future research on cerebral infarction.

There is a need to shift from a focus on “symptom notification” to “abnormal identification and calibration.” During the appraisal assessment phase, patients often tend to “normalize” early or atypical symptoms of cerebral infarction, integrating them into an explanatory framework that includes fatigue, cold, or natural aging. This tendency results in delays in taking appropriate action. Consequently, traditional health education that emphasizes symptom lists proves inadequate in effectively promoting timely responses. In practice, it is essential to enhance contextualized information regarding “when not to wait” for high-risk groups, such as the older adult(s), rural residents, and patients with chronic diseases. Utilizing specific case examples can help clarify which physical changes should not be dismissed as mere daily discomfort, thereby encouraging patients to recognize health threats earlier and initiate appropriate responses.

Correcting cognitive biases and optimizing doctor-patient communication is essential. During the illness delay stage, optimism bias and an overestimation of self-regulation abilities lead patients to adopt a “wait and see” approach, which may reinforce maladaptive self-processing even in the absence of symptom relief. Concurrently, some patients harbor systematic doubts regarding medical advice, such as recommendations for examinations and referrals, which results in confirmation bias. In this context, clinical practice can benefit from the “nudge” strategy in behavioral science by implementing personalized risk communication that optimizes the information presentation framework (37). For instance, converting the abstract concept of a “treatment time window” into a specific and tangible number of neuron losses (loss frame) or juxtaposing successful cases of timely medical intervention with adverse outcomes following delays (social norms and outcome framework) can effectively counteract patients’ cognitive inertia. This approach aids in establishing realistic expectations regarding disease progression and treatment efficacy while preventing the misinterpretation of “uncertainty” as “not serious.” Ultimately, such strategies may reduce delayed decision-making stemming from cognitive biases.

Reducing structural costs while addressing concerns regarding social consequences is essential. During the medical utilization phase, financial pressures, familial obligations, and fears of becoming a burden often overshadow patients’ health needs. Even when patients acknowledge the efficacy of a medical intervention, apprehensions about long-term financial implications and family impact may cause delays in their decision-making. Consequently, it is imperative at the policy level to enhance medical insurance coverage for the long-term management and rehabilitation phases of cerebral infarction. Additionally, developing a tiered diagnosis and treatment framework, along with a community rehabilitation support system, can alleviate patients’ subjective expectations of “long-term unbearable costs.” Furthermore, clinical decision support should more comprehensively consider the patient’s familial role and social circumstances, assisting them in establishing a feasible balance between health needs and family responsibilities.

Establishing a supportive social environment is essential for reducing stigma and fatalism. Research indicates that stigma and fatalistic attitudes significantly hinder proactive medical-seeking behavior, particularly among older adult(s) patients. Effective interventions should extend beyond the individual level, emphasizing communication at both community and social levels. It is crucial to challenge the cultural narrative that frames neurological dysfunction as a “personal failure” or a matter of “fate.” Additionally, reshaping the societal perception of timely medical treatment is necessary, portraying it as a positive action that sustains family dynamics and enhances personal social value, rather than as a burden on others.

## 7 Research limitations and future directions

Although this study systematically revealed the staged decision-making mechanism of patients with cerebral infarction delaying medical treatment through a mixed methods method, there are still several limitations: First, the sample of this study came from tertiary hospitals in the more economically developed areas of eastern China. This can weaken the interference of insufficient access to medical resources, but it may also limit the extrapolation of research conclusions to areas with relatively scarce medical resources. In such resource-limited settings, structural barriers (e.g., transportation difficulties, limited healthcare facilities, and greater economic constraints) may interact with the cognitive mechanisms identified in this study, and could even become the dominant drivers of prehospital delay. Future research should expand the sample size and engage in multi-center collaborations among primary medical institutions across various regions. This approach will facilitate the identification of common factors influencing prehospital delays and allow for the testing of the situational stability of the mechanisms proposed in this article. Second, this study reconstructed patients’ decision-making processes through retrospective interviews, which may be subject to recall bias. Future research can use prospective or longitudinal designs to dynamically track the evolution of symptom perception, cognitive assessment, and behavioral adjustment among high-risk groups, thereby more precisely characterizing the time structure of delayed medical treatment. Third, this study did not conduct a systematic comparison of the heterogeneity of delay mechanisms across gender, age, or family structure. In view of the important role of social roles and family relationships in health decision-making, future research can further introduce a hierarchical or interactive perspective to explore the differential performance of staged decision-making mechanisms in different groups of people.

## 8 Conclusion

This study reveals that prehospital delay in cerebral infarction follows a dual-pathway process shaped by cognitive-behavioral and structural factors. Key predictors—including perceived control, family norms, affective attitudes, and past health habits—influence delay even among patients aware of symptom severity. The persistence of cognitive-behavioral disconnection highlights the need for integrated interventions targeting both decision-making pathways and contextual barriers. Future research should test staged strategies that jointly address cognitive restructuring and structural support to improve timely care-seeking.

## Data Availability

The raw data supporting the conclusions of this article will be made available by the authors, without undue reservation.
