# Supplementary material for: From symptom onset to care-seeking: a process-oriented mixed-methods study of prehospital delay in cerebral infarction
Source: Front Public Health. 2026 Jul 3;14:1823472. doi: 10.3389/fpubh.2026.1823472 (PMC13376248; doi:10.3389/fpubh.2026.1823472)
Supplement: Supplementary file 1 [file Table_1.doc]

Appendix

Table 2A. Spearman correlations between BIPQ dimensions and delay time (N = 68)

| Variable | Item1 | Item2 | Item3 | Item4 | Item5 | Item6 | Item7 | Item8 | Delay time |
| --- | --- | --- | --- | --- | --- | --- | --- | --- | --- |
| Item1 | 1.000 | .295* | .012 | .053 | .581** | .165 | .043 | .636** | -.058 |
| Item2 | .295* | 1.000 | .320** | .226 | -.030 | .124 | .211 | .269* | .143 |
| Item3 | .012 | .320** | 1.000 | .163 | -.053 | .197 | .084 | .153 | -.015 |
| Item4 | .053 | .226 | .163 | 1.000 | .194 | .324** | .011 | .197 | .127 |
| Item5 | .581** | -.030 | -.053 | .194 | 1.000 | .325** | .074 | .541** | -.111 |
| Item6 | .165 | .124 | .197 | .324** | .325** | 1.000 | .134 | .270* | -.045 |
| Item7 | .043 | .211 | .084 | .011 | .074 | .134 | 1.000 | .123 | .228 |
| Item8 | .636** | .269* | .153 | .197 | .541** | .270* | .123 | 1.000 | .027 |
| Delay time | -.058 | .143 | -.015 | .127 | -.111 | -.045 | .228 | .027 | 1.000 |

*Note.* *p < 0.05; **p < 0.01 (two-tailed).

Table 2B. Comparison of BIPQ dimensions between short-delay (≤7 days) and long-delay (>7 days) groups

| BIPQ dimension | | Mann-Whitney U | | Wilcoxon W | | Z | | Sig. (2-tailed) | |
| --- | --- | --- | --- | --- | --- | --- | --- | --- | --- |
| Item1 | 545.000 | | 1041.000 | | -0.356 | | 0.722 | |  |
| Item2 | 509.500 | | 1212.500 | | -0.835 | | 0.404 | |  |
| Item3 | 570.500 | | 1273.500 | | -0.038 | | 0.970 | |  |
| Item4 | 479.500 | | 1182.500 | | -1.208 | | 0.227 | |  |
| Item5 | 526.500 | | 1022.500 | | -0.593 | | 0.553 | |  |
| Item6 | 539.500 | | 1242.500 | | -0.435 | | 0.664 | |  |
| Item7 | 474.500 | | 1177.500 | | -1.237 | | 0.216 | |  |
| Item8 | 508.500 | | 1211.500 | | -0.806 | | 0.420 | |  |

*Note.* Short-delay group: ≤7 days (n = 37); long-delay group: >7 days (n = 31).

Table 2C. Stepwise linear regression analysis for predictors of delay time

| Variable | B | SE | β | t | p | 95% CI |
| --- | --- | --- | --- | --- | --- | --- |
| (Constant) | 0.491 | 0.203 |  | 2.422 | 0.018 | 0.086, 0.896 |
| Item7 | 0.076 | 0.037 | 0.245 | 2.049 | 0.044 | 0.002, 0.150 |

*Note.* Dependent variable: Log delay time. R² = 0.060, adjusted R² = 0.046, F(1, 66) = 4.199, p = 0.044.
